# Comprehensive survey of human brain microRNA by deep sequencing

**DOI:** 10.1186/1471-2164-11-409

**Published:** 2010-06-30

**Authors:** Ning-Yi Shao, Hai Yang Hu, Zheng Yan, Ying Xu, Hao Hu, Corinna Menzel, Na Li, Wei Chen, Philipp Khaitovich

**Affiliations:** 1 Partner Institute for Computational Biology, 320 Yueyang Road, 200031, Shanghai, China; 2 Graduate School of Chinese Academy of Sciences, 19 Yuquan Road, 100039, Beijing, China; 3 Max Planck Institute for Molecular Genetics, Ihnestrasse 63-73, D-14195 Berlin, Germany; 4 Max-Delbrück-Centrum für Molekulare Medizin, Berlin Institute for Medical Systems Biology, Robert-Rössle-str 10, D-13092, Berlin, Germany; 5 Max Planck Institute for Evolutionary Anthropology, Deutscher Platz 6, D-04103 Leipzig, Germany

## Abstract

**Background:**

MicroRNA (miRNA) play an important role in gene expression regulation. At present, the number of annotated miRNA continues to grow rapidly, in part due to advances of high-throughput sequencing techniques. Here, we use deep sequencing to characterize a population of small RNA expressed in human and rhesus macaques brain cortex.

**Results:**

Based on a total of more than 150 million sequence reads we identify 197 putative novel miRNA, in humans and rhesus macaques, that are highly conserved among mammals. These putative miRNA have significant excess of conserved target sites in genes' 3'UTRs, supporting their functional role in gene regulation. Additionally, in humans and rhesus macaques respectively, we identify 41 and 22 conserved putative miRNA originating from non-coding RNA (ncRNA) transcripts. While some of these molecules might function as conventional miRNA, others might be harmful and result in target avoidance.

**Conclusions:**

Here, we further extend the repertoire of conserved human and rhesus macaque miRNA. Even though our study is based on a single tissue, the coverage depth of our study allows identification of functional miRNA present in brain tissue at background expression levels. Therefore, our study might cover large proportion of the yet unannotated conserved miRNA present in the human genome.

## Background

MicroRNA (miRNA) are a specific class of small RNA involved in posttranscriptional gene regulation in a wide variety of species. Typical miRNA are single-stranded RNA molecules approximately 22 nucleotides in length [1]. In animals, miRNAs are cut from a longer, single-stranded RNA precursor that forms a hairpin loop structure by two endonucleases, Drosha and Dicer, assisted by auxiliary protein factors [2]. Mature miRNA function as a component of an RNA-protein complex known as RNA-Induced Silencing Complex (RISC). As a part of the complex, miRNA guides it to specific gene targets through base-pairing interaction between the miRNA seed region and a complementary sequence in the mRNA. In humans and other animals, the seed region normally extends from the second to eighth positions of mature miRNA [1-3]. Within mRNA, miRNA target sites are mainly located within its 3'UTR, although a few miRNA binding sites are found in 5'UTR and the coding region [4,5]. Interaction between RISC and mRNA normally leads to somewhat moderate repression of gene expression, usually through both translation inhibition and mRNA degradation [2]. Such miRNA-assisted gene repression was shown to play an essential role in many development and differentiation pathways across species [6].

From their discovery in 1990s, numbers of identified miRNA are increasing rapidly [7-9]. At present, there are 896 miRNA annotated in the human genome (miRBase Version 14.0). The main criteria for miRNA identification are: (i) presence of a sequence motive capable of forming a hairpin structure over at least 20 nucleotides devoid of large loops and bulges, and (ii) expression of a distinct RNA sequence approximately 22 nucleotide in length, originating from one of the hairpin arms [10]. Within the human genome, close to 450,000 regions could form long unbranched hairpin structures which, if transcribed and processed, can result in mature miRNA [11]. Some of these sequences are not conserved across species and tend to be expressed at low levels [12,13]. Such sequences, although capable of becoming functional miRNA over long evolutionary time [14], might not have any immediate functional significance [15]. Still, as our knowledge of human miRNA expression across tissues and ontogenetic stages is incomplete, the full variety of human functional miRNA is not known [16]. Specifically, many evolutionary conserved sequences capable of forming miRNA precursor hairpins still lack evidence of mature miRNA expression. Further, highly expressed and fast evolving functional miRNA that are lacking sequence conservation might still be missed. Here, we attempt to partially fill this gap by comprehensive characterization of human and rhesus macaque miRNA transcriptome in a specific brain region, namely the dorsolateral prefrontal cortex, using deep high-throughput sequencing.

## Results and Discussion

### Small RNA sequencing and characterization

To get a comprehensive view of small RNA expressed in human and rhesus macaque dorsolateral prefrontal cortex, we sequenced RNA fraction with sizes from 18 to 28 nucleotides (nt) in 12 human and 12 rhesus macaque healthy male individuals, using Illumina Genome Analyzer. To obtain maximal representation of small RNA, we included samples covering most of the human and rhesus macaque lifespans: from birth to 98 years of age for humans, and from birth to 28 years of age for macaques (Additional file 1: Table S1). Combining these samples, we obtained a total of 76,565,933 sequence reads, corresponding to 909,917 unique sequences in humans, and a total of 95,326,968 reads, corresponding to 970,340 unique sequences in macaques. Allowing no mismatches and only considering the sequences represented by at least two reads, 55,061,969 sequence reads could be mapped to the human genome and 69,315,085 sequences reads to the rhesus macaque genome (Additional file 1: Tables S2, S3).

**Figure 1:**
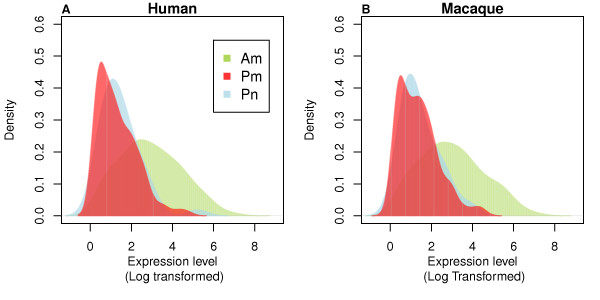
**Expression levels of conserved annotated and putative miRNA**. Distribution of expression levels (base-10 logarithm transformed) of conserved annotated miRNA (Am/green), conserved putative miRNA, excluding the ones originating from ncRNA (Pm/red), and conserved putative miRNA originating from ncRNA (Pn/blue) in humans (**A**) and rhesus macaques (**B**).

Out of all sequence reads that map to the human genome, 97.2% correspond to 602 annotated human miRNA. Similarly, in rhesus macaques, 97.9% of all mapped reads correspond to 493 macaque orthologs of annotated human miRNA [17]. In agreement with previous studies [18,19], miRNA are expressed in brain at a broad concentration range, spanning more than six orders of magnitude (see Figure 1 and Additional file 1: Figure S1). Consequently, as much as 88% of all sequence reads mapped to miRNA correspond to 20 highly expressed miRNA in both humans and rhesus macaques (Table 1). On the other hand, many miRNA are represented by only a few sequence reads. Despite the low abundance, most of these miRNA are highly conserved among mammals and some, such as miR-29, miR-103, miR-101, were shown to function in other tissues [20-23]. Low expression of these miRNA in our dataset may be due to the following reasons: they are expressed in a limited number of brain cells, or they play no functional role in the prefrontal cortex and are expressed at the "background" transcription level. Importantly, this result indicates that novel human and macaque miRNA that do not function in postnatal prefrontal cortex or functional in a limited set of cells can still be detected in our dataset at low expression levels.

**Table 1:** Top 40 highly expressed annotated miRNA found in brain transcriptomes of humans and rhesus macaques using Illumina sequencing

Human		Rhesus macaque*	
**Annotated miR**	**Mapped reads**	**Annotated miR**	**Mapped reads**

hsa-let-7f	18832757	hsa-let-7f	20521904

hsa-let-7g	7578064	hsa-let-7g	12944454

hsa-let-7a	5117974	hsa-let-7c	5671467

hsa-let-7c	4516955	hsa-let-7a	5531680

hsa-mir-128	1561400	hsa-mir-128	2039653

hsa-let-7b	1484735	hsa-mir-103	2023922

hsa-mir-29a	1321992	hsa-let-7b	1986248

hsa-mir-103	1303040	hsa-mir-29a	1584566

hsa-mir-101	857034	hsa-mir-107	1295564

hsa-mir-1	797282	hsa-mir-1	861800

hsa-mir-107	768588	hsa-let-7i	770838

hsa-mir-140-3p	712479	hsa-mir-140-3p	769912

hsa-mir-124	608149	hsa-mir-101	754183

hsa-let-7i	584441	hsa-let-7e	750264

hsa-let-7e	562093	hsa-mir-124	727892

hsa-mir-340	394365	hsa-mir-7	717781

hsa-mir-143	377898	hsa-let-7d	658387

hsa-mir-7	371940	hsa-mir-221	410955

hsa-mir-9	349404	hsa-mir-181a	402205

hsa-mir-181a	270803	hsa-mir-340	328441

hsa-let-7d	264273	hsa-mir-222	325038

hsa-mir-26a	251367	hsa-mir-125b	305229

hsa-mir-125b	221344	hsa-mir-320a	297817

hsa-mir-219-2-3p	196671	hsa-mir-143	295807

hsa-mir-29c	195287	hsa-mir-26a	287286

hsa-mir-9*	162117	hsa-mir-29c	253594

hsa-mir-221	149379	hsa-mir-9	252327

hsa-mir-221*	144201	hsa-mir-191	242534

hsa-mir-330-3p	143784	hsa-mir-221*	241349

hsa-mir-191	143704	hsa-mir-9*	224604

hsa-mir-26b	138381	hsa-mir-383	206070

hsa-mir-99a	123383	hsa-mir-199a-3p	195938

hsa-mir-21	122560	hsa-mir-99a	194879

hsa-mir-192	108801	hsa-mir-185	194288

hsa-mir-30a	106646	hsa-mir-99b	182985

hsa-mir-222	97768	hsa-mir-219-2-3p	175644

hsa-mir-199a-3p	95642	hsa-mir-330-3p	172866

hsa-mir-199b-3p	95639	hsa-mir-181b	170769

hsa-mir-99b	92083	hsa-mir-485-5p	128293

hsa-mir-320a	91968	hsa-mir-30a	125186

* miRNA annotation of rhesus macaque is based on human annotation by mapping of macaque miRNA precursors to the human genome by the reciprocal LiftOver.

### Novel miRNA identification

To identify potential novel miRNA, we further analyzed the 1,494,224 human and 1,421,666 rhesus macaque sequence reads that remained after excluding the reads mapped to the annotated miRNA. During miRNA maturation, the pre-miRNA hairpin can produce two different mature miRNA, one from each of the hairpin arms. While 235 miRNA precursors are known to produce two mature miRNA, 426 are annotated to produce just one (miRBase 14.0). Therefore, we first searched for yet undiscovered miRNA originating from the known precursors. Using this approach, 96 and 73 such miRNA, each supported by at least two sequence reads, can be identified in humans and rhesus macaques, respectively. While these miRNA would be commonly classified as miRNA-star (miRNA*), a low expressed by-product of miRNA generation, some of them are highly expressed, both relatively and absolutely. Specifically, out of 96 novel human miRNA*, 33 are expressed higher than their annotated counterpart originating from the same precursor, and 4 are expressed at copy number greater than 3,000. Thus, many of the 96 novel human miRNA* might be as functional as their annotated miRNA counterparts.

Next, we identified putative miRNA originating from novel precursors using two established approaches. In the first approach, we used RNALfold [24], to identify transcribed genomic regions that can form stable non-branching hairpin structures containing at least 20 basepairs within hairpin stem [10]. We then used miPred [25], a Random Forest-based classification algorithm, to identify hairpins with sequence features characteristic to precursors of known human miRNA. We used hairpins derived from exon regions as a negative set (see Methods). Out of 602 known human and 493 known macaque miRNA represented in our dataset, 516 (85.7%) and 414 (84.0%), respectively, passed this identification pipeline, indicating high sensitivity of this method (see Additional file 1: Table S4). Applying it to the rest of the dataset, we identify 1,388 putative human miRNA, each represented by at least two sequence reads. Among these miRNA, 62 originate within other annotated ncRNA. For the rhesus dataset, we find 1,052 putative miRNA, 30 originating from other annotated ncRNA (Additional file 1: Tables S5, S6, Figure S2).

In the second approach, we used the miRDeep algorithm to identify patterns of short sequence reads characteristic to miRNA precursors [18]. As this approach requires substantial read-density, miRNA represented by few sequence reads will not be identified. Consequently, out of 602 known human miRNA represented in our dataset, only 211 (35.0%) passed the miRDeep algorithm with high confidence level criteria. Using the same criteria, miRDeep identifies 65 and 108 putative novel human and rhesus macaque miRNA, respectively. Out of these miRNA, 51 and 76, respectively, overlap with putative miRNA predicted by the first approach (Additional file 1: Tables S5, S6, Figure S2). This overlap is much greater than expected by chance (hypergeometric test, p ≪ 0.001), even though the two miRNA prediction methods employ very different miRNA identification strategies.

### Novel miRNA sequence conservation

**Figure 2:**
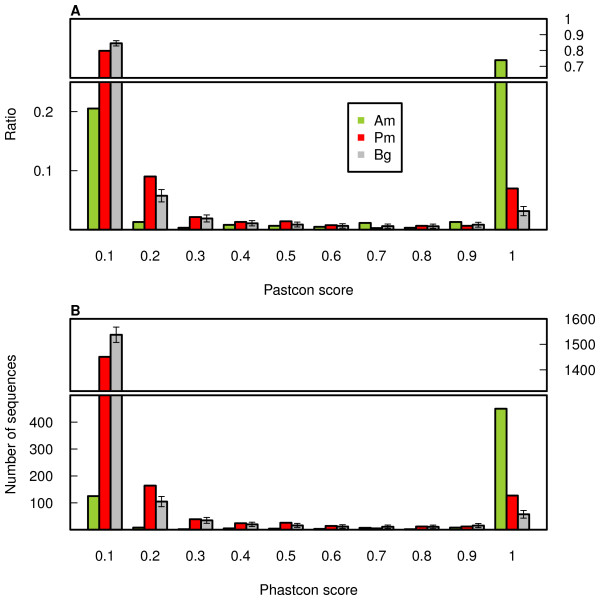
**Conservation of human annotated and putative miRNA**. The distribution of average sequence conservation scores (phastCon scores) based on alignment of 18 placental mammalian genomes within annotated human miRNA (Am/green), novel putative miRNA that passed our pipeline, excluding the ncRNA-derived miRNA (Pm/red), and the background phastCon scores distribution (Bg/grey) based on 1,000 simulations (see Methods for details). The error bars show 95% confidence interval of the background phastCon scores distribution. Shown are the proportions (**A**) and the numbers (**B**) of miRNA in each of the 10 phastCon score bins (between 0 and 1, from the least conserved to the most conserved, divided into equal intervals).

As the function of miRNA depends on their sequence, functional miRNA are expected be conserved. Among the total of 1,498 putative human miRNA identified by either of the two approaches, 65 map within genomic regions corresponding to known non-coding RNA (ncRNA), such as tRNA, snRNA, or snoRNA. Such putative miRNA might be conserved on the DNA sequence level as a part of longer functional transcripts and, therefore, were excluded from the conservation analysis. To assess sequence conservation of the remaining 1,433 putative novel human miRNA (Additional file 1: Tables S5), we used phastCon scores based on the genomic sequence comparison among 18 placental mammals [26]. We find that 132 identified putative human miRNA are highly conserved among mammals (average phastCon score ≥0.8), while 73 would be expected to score as high by chance (simulations, p < 0.001) (Figure 2). Further, 67 (50.7%) of these conserved miRNA are also expressed in rhesus macaques and pass our miRNA-identification procedure, while 29.5% are expected by chance (simulations, p < 0.001). In rhesus macaques, we find additional 65 putative conserved miRNA, all of which can also be found in the human genome, but are not expressed in the human dataset.

Certainly, the proportion of highly conserved miRNA is much smaller for the putative novel miRNA than for the annotated miRNA (Table 2). This, however, is due to the fact that many annotated miRNA were first identified based on their sequence conservation [1,8,12].

**Table 2:** Expression levels and the origins of the top 20 highly expressed putative novel miRNA identified in the human and rhesus macaque brain transcriptomes

Human			
**Mature sequences**	**Mapped reads**	**Mapped region annotation**	**Novel miRNA-star**

AUCAUACAAGGACAAUUUCUUU	56157		hsa-mir-539*

AAUCAUUCACGGACAACACUUU	26634		hsa-mir-382*

ACCUUGGCUCUAGACUGCUUACU	13153		hsa-mir-212*

UGGGCUGUAGUGCGCUAUGCCGAU	8775	MER11B	

GCGUUGGUGGUAUAGUGG	5183	tRNA-Gly-GGA	

CAGGCAGUGACUGUUCAGACGUC	3586		

GUCUCUGUGGCGCAAUCGGU	3467		

UGUAGGGAUGGAAGCCAUGAAA	3170		hsa-mir-135a*

UGGGCUGUAGUGCGCUAUGCC	1437	7SLRNA	

AGUUGGUCCGAGUGUUGUGGGUUAUU	1413	HY5	hsa-mir-1975*

AAUCUGAGAAGGCGCACAAGGUUU	958	LTR16D	

AGAGGUUUUCUGGGUUUCUGUUU	832		hsa-mir-329*

AAUGUGUAGCAAAAGACAGA	746		hsa-mir-511*

AUCCCCAGAUACAAUGGACAAU	719		

GGAGGAACCUUGGAGCUUCGGCA	695		

GCUCUGACUUUAUUGCACUACU	686		hsa-mir-301a*

AUAUACAGGGGGAGACUCUUAU	636		hsa-mir-1185*

UGGUCGACCAGUUGGAAAGUAAU	601		hsa-mir-412*

AGGCAUUAGAUUCUCAUUAGGA	562	MER1A, MER1B	

AUAGGACUCAUAUAGUGCCA	531		

			

Rhesus macaque*			

**Mature sequences**	**Mapped reads**	**Mapped region annotation**	**Novel miRNA-star**

CCCCCCACUGCUAAAUUUGACUGGCUU	243332	HY4	

AAUCAUUCACGGACAACACUUU	33573		hsa-mir-382*

GAGAGAUCAGAGGCGCAGAGU	18711		

AUCAUACAAGGACAAUUUCUUU	16465		hsa-mir-539*

AAGUUUCUCUGAAUGUGUAGA	12107	U3	

CUGUGGUUCCUGUAUGAAGACA	11261		

UGUAGGGAUGGAAGCCAUGA	5546		hsa-mir-135a*

ACUGGACUUGGAGUCAGAAG	5503	MIRb	

GCAUUGGUGGUUCAGUGGUAGAAUUC	4916	AluMacYa3	

GGGGGCCGAUACACUGUACGAGA	3972		hsa-mir-128*

GUAAUGGUUAGCACUCUGG	2008	AluSx, Zaphod	

AAUAUACAGGGGGAGACUCUUAU	1912		hsa-mir-1185*

UGGUCGACCAGUUGGAAAGUAAU	1526		hsa-mir-412*

GCUCUGACUUUAUUGCACUACU	1476		hsa-mir-301a*

UGAGUCUGUAAGAAAAGAGGAG	1472		

AAUGUGUAGCAAAAGACAGAAU	1073		hsa-mir-511*

AGGGACUUUUGGGGGCAGAUGUGU	1068		hsa-mir-365*

GGAGACUGAUGAGUUCCCGGGA	922		hsa-mir-873*

CAACAAAUCACAGCCGGCCUCA	919		hsa-mir-7*

AAUCUGAGAAGGCGCACAAGGUUU	852	LTR16D	

* miRNA annotation of rhesus macaque used for labelling of the identified novel miRNA-star sequences is based on human annotation by mapping of macaque miRNA precursors to the human genome by the reciprocal LiftOver.

**Figure 3:**
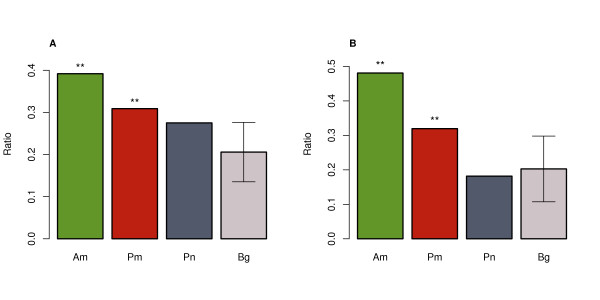
**Target site conservation excess of annotated and putative miRNA**. The proportion of seed families with target site conservation excess (p < 0.05) among annotated conserved miRNA (Am/green), putative conserved miRNA excluding ncRNA-derived miRNA (Pm/red), and ncRNA-derived putative conserved miRNA identified in human dataset (**A**) and overlapping between human and rhesus datasets (**B**). The background proportion (Bg/grey) was calculated by 1,000 random simulations of seed region sequences (see Methods for details). The error bars show 95% confidence interval of the simulations. The stars above the bars (**) indicate significance level based on the simulation results.

Still, conservation of putative miRNA sequences alone is not a sufficient proof of their functionality. Most mammalian mRNAs recognize their target genes through basepairing interaction between 7-mer sequence at the 5'-end of miRNA, known as a seed region, and a complimentary sequence at the target's 3' UTR. Thus, for many annotated conserved miRNA, the complementary target sequences show increased conservation as well [27-29]. Based on this property, we can further assess functionality of conserved putative novel miRNA. As several miRNA may share the same seed sequence, we combined all conserved miRNA into families, each containing a unique seed. This resulted in 342 annotated and 123 putative human miRNA families. For each family, we determined a target conservation score using a previously published approach [30]. Briefly, the method tests whether 3' UTR sequences complimentary to the chosen miRNA seed region are more conserved among mammals than the control sequences. The control sequences are 3' UTR sequences complimentary to a scrambled seed region and occurring within 3' UTRs at a similar frequency (±10%). Using this method, 39.1% (134 out of 342) of annotated miRNA families and 30.9% (38 out of 123) of putative miRNA families show significant excess of conserved target sequences (p < 0.05). As the test is sensitive to the number of potential target sites, miRNA targeting few genes may not pass the significance cut-off. This could explain the low sensitivity of the test for both annotated and novel miRNA families. Still, for both annotated and novel miRNA, the proportion of families with significant excess of conserved target sequences is greater than expected by chance (simulations, p < 0.001 and p = 0.005, respectively) (Figure 3). Thus, a sizable proportion of conserved putative miRNA identified in this study is likely to be functional.

### Novel miRNA expression

The vast majority of putative miRNA identified in this study, both conserved and non-conserved, are expressed at low levels (Figure 1 and Additional file 1: Figure S1). Many of non-conserved putative miRNA may result from spurious processing of transcribed hairpin structures by Drosha and Dicer and have no immediate functional significance, as suggested previously [12]. Still, some show relatively high expression: 3 out of 1,310 non-conserved putative human miRNA are represented by more than a 1,000 sequence reads, and 32 - by more than a 100 (Table 3). Some of these putative miRNA (3/3 and 18/32) originate from repetitive genomic regions (Additional file 2: Table S7). Although some miRNA originating within repeats were suggested to be functional [31,32], proving this would require further evidence. Conserved putative miRNA, on the other hand, might be functional even when present at low levels in our dataset, as they may function in specific cell types, tissues, and/or ontogenetic stages not captured by our study.

**Table 3:** Expression levels and the origins of the top 20 highly expressed non-conserved putative novel miRNA identified in the human brain transcriptomes

Mature sequences	Mapped reads	Mapped region annotation
UGGGCUGUAGUGCGCUAUGCCGAU	8,775	MER11B

GCGUUGGUGGUAUAGUGG	5,183	tRNA-Gly-GGA

UGGGCUGUAGUGCGCUAUGCC	1,437	7SLRNA

AAUCUGAGAAGGCGCACAAGGUUU	958	LTR16D

AAUGUGUAGCAAAAGACAGA	746	

GGAGGAACCUUGGAGCUUCGGCA	695	

AGGCAUUAGAUUCUCAUUAGGA	562	MER1A

AUAGGACUCAUAUAGUGCCA	531	

UGGUAGAAUUCUCGCCUGCC	497	tRNA-Gly-GGY

CUUCUGCAUGGACUAGACAUUG	429	

AGAGGUGUAGAAUAAGUGGGAG	309	LSU-rRNA_Hsa

AUAUACAGGGGGAGACUCUCAU	297	

CAAAGACUGCAAUUACUUUUG	269	MADE1

CAAAAGUGAUUGUGGUUUUUGC	258	MADE1

CAGGGCUGGCAGUGACAUGGGU	248	

UGGUAGAAUGACAGGCCACG	242	

AGGGGGCGGGCUCCGGCG	240	

AGAAGUUUCUCUGAACGUGUAU	231	U3

AGAAGUUUCUCUGAACGUGUAA	186	U3

CUGACACUUCUCAGAAUUCUUA	166	

### Novel miRNA originating from other ncRNA

Small RNA originating from highly expressed transcripts, such as known ncRNA, might represent degradation products of longer transcripts and, therefore, are commonly excluded from the miRNA identification studies [8]. Recent work, however, has shown that some small RNA originating from other ncRNA can function as miRNA [14]. Among all putative human miRNA sequences identified in this study, 65 are contained within known ncRNA transcripts, 41 of them conserved among placental mammals. Similarly, in rhesus macaques we find 32 putative miRNA originated from other ncRNA, 22 of them conserved among placental mammals. Out of a total of 41 conserved putative human miRNA, 40 originate from five ncRNA types: C/D Box snoRNA, H/ACA Box snoRNA, scaRNA, snRNA, and tRNA (Additional file 3: Table S8).

To assess whether these ncRNA fragments could function as miRNA, we analyzed the sequence conservation of their predicted target sites within 3' UTRs of protein-coding genes. We find that out of 41 seed families derived from 41 conserved putative human miRNA, 11 (26.8%) show significant excess of conserved target sequences (p < 0.05) (Figure 3 and Additional file 1: Figure S4). This proportion is higher than the background one (20.6%), but not significantly (p = 0.26). Thus, overall we do not find a support for ncRNA-derived putative miRNA functionality.

Still, the highest expressed ncRNA-derived putative miRNA identified in our study comes from a member of scaRNA family, ACA45 RNA, previously shown to give rise to a functional mature miRNA [14] (Additional file 1: Figures S3 and S5A). The seed region of this miRNA shows marginally significant excess of conserved target sites (p = 0.067), further supporting its functional significance. Overall, out of all 41 ncRNA-derived conserved human putative miRNA, 11 show significant enrichment of conserved target sites (p < 0.05). Among them is another highly expressed putative miRNA derived from a member of scaRNA family, ACA47 RNA (Additional file 1: Figures S3 and S5B). This putative miRNA, along with miRNA originating from ACA45 RNA, were shown to co-precipitate in complex with human Ago proteins, indicating its potential functionality [14,33].

Notably, out of 41 ncRNA-derived putative miRNA identified in our study, another 11 miRNA have significantly fewer conserved target sites in all annotated mRNA 3' UTRs than expected by chance (p < 0.05). This indicates long-term selective pressure on 3' UTR sequences to avoid targeting by these putative miRNA. Such a situation may arise when generation of miRNA-like molecules from these ncRNA has a detrimental effect on gene expression regulation. At the same time, functional constraints on ncRNA sequences conserve features leading to hairpin recognition by miRNA processing machinery.

Supporting this notion, the two putative miRNA with the lowest target conservation are highly expressed in our dataset (Additional file 1: Figure S3). One of them originates from H/ACA box snoRNA and another from tRNA-Ile. Interestingly, a recent study has demonstrated that in mouse embryonic stem cells, tRNA-Ile can form an alternative long hairpin structure, recognized by Dicer, instead of the typical "clover leaf" structure [34]. In humans, the tRNA-Ile can also form an alternative long hairpin structure with low free energy (MFE of a long hairpin structure is -50.4 kcal/mol and the clover leaf is -44.5 kcal/mol). Processing of such a long hairpin structure could generate miRNA-like molecules detected in our study (Additional file 1: Figure S5C). Similarly, ACA18 RNA snoRNA forms a long hairpin structure that could potentially be recognized by miRNA processing machinery (Additional file 1: Figure S5D). Small RNA resulting from this processing, however, appear to play deleterious roles in gene regulatory networks, rather than being incorporated into them as functional miRNA.

## Conclusions

In this study, we take advantage of the high throughput sequencing technology to obtain a comprehensive view of small RNAs expressed in the human and rhesus macaque brain cortex. Sequencing deeply, we detect both miRNA that function in brain and miRNA expressed as "background" transcripts. This gives us an opportunity to identify putative novel miRNA that may function in tissues and ontogenetic stages not included in our analysis.

It has to be noted, however, that miRNA expression levels determined in our study may not fully reflect actual miRNA concentrations within the tissue. As shown previously, the results of miRNA concentrations estimates tend to vary substantially between high-throughput sequencing and microarrays [35], as well as miRNA between different high-throughput sequencing methodologies [36,37]. Thus, although estimated miRNA expression levels do, to an extent, reflect actual miRNA concentrations, the technical biases of the high-throughput sequencing technology used limit our ability when investigating expression level correlations and relationship between miRNA expression and target site abundance.

Another limitation of our study is that our analysis of novel miRNA functionality. It is based on the target site conservation and, therefore, can only be applied to evolutionary conserved miRNA. Thus, fast evolving miRNA will not be classified as functional in this analysis. Nonetheless, since most of non-conserved putative miRNA identified in our study are low-expressed, the numbers of potential functional fast-evolving miRNA present in the human brain are likely to be small. The few identified highly expressed non-conserved putative miRNA tend to originate within repeated regions, making it difficult to infer their functionality.

We further identify 41 and 22 conserved putative miRNA originating from ncRNA in humans and rhesus macaques, respectively. Some of these putative miRNA show significant excess of conserved target sites and might function as conventional miRNA. Others, however, might interfere with the existing regulatory networks, resulting in target avoidance.

Finally, in humans and macaques, we identify 197 putative novel miRNA, highly conserved among mammals, with 68 detected in both species. Although most of these putative miRNA are expressed at low levels in our dataset, they show significant excess of conserved target sites in the 3'UTRs of protein-coding genes. This indicates a substantial proportion of identified putative miRNA may indeed represent functional miRNA. Given the sequencing depth of our study, it is likely that these novel miRNA capture most of the yet unidentified conserved human miRNA repertoire.

## Methods

### Small RNA library preparation

Human tissues were obtained from the NICHD Brain and Tissue Bank for Developmental Disorders at the University of Maryland, Baltimore, MD, USA, and the Chinese Brain Bank Center, Wuhan, China. Informed consent for use of the human tissues for research was obtained in writing from all donors or their next of kin. Human subjects were defined as normal controls by brain bank pathologists. No research subject had a prolonged agonal state. Rhesus (Macaca mulatta) brain samples were obtained from the Suzhou Experimental Animal Center, Suzhou, PR China.

In this study, samples were obtained from the anterior portion of superior frontal gyrus (Brodmann Area 9) of 12 humans (ages from 0 to 98 years) and 12 Rhesus monkeys (0-28 years) (Additional file 1: Table S1). For all samples, cortical dissections contained approximately 2:1 volume ratio of grey matter to white matter.

Total RNA was isolated from the frozen cortex tissue using Trizol (Invitrogen, USA) protocol with no modifications. Low molecular weight RNA was isolated by electrophoresis, ligated to the adapters, amplified, and sequenced following the Small RNA preparation protocol (Illumina, USA) with no modifications.

### Reads mapping and annotation

We trimmed the adaptor at the 3-prime of the 36 nucleotide-long sequence reads allowing three mismatches between the adapter and read sequences. We further removed low complexity sequences using mDust [38]. Further, only sequences of length 18-28 nt represented by at least two sequence reads were used in mapping procedure (Additional file 1: Figure S2). Except for the test of the mapping procedure fidelity (Additional file 1: Table S3), we mapped the sequences to the human genome (Hg 18) and the rhesus macaque genome (rheMac2) using ELAND (version 0.3.0) [39], allowing no mismatches.

Mapped sequence reads were annotated as miRNA, ncRNA, repeat, exon, intron, or intergenic if at least half of its sequence fell into the corresponding genome annotation region. Genomic annotation of repeat and ncRNA (except miRNA and piRNA) was downloaded from the UCSC Genome Browser [40]. The ncRNA annotation of rhesus macaques was based on the reciprocal LiftOver of the UCSC human ncRNA annotation. The detail annotation of snoRNA was downloaded from snoRNABase [41]. The positions of human miRNA were downloaded from miRBase (version 14.0) [10,42-45]. The miRNA annotation in rhesus macaques was based on reciprocal alignment of human miRNA precursors by LiftOver [46-48]. The exon and intron annotations of protein-coding RNA were downloaded from Biomart Project Website [49].

### microRNA prediction

We used RNALfold to detect the locally stable secondary substructures in the genomic region from 100 nt upstream to 100 nt downstream from all of the mapped sequence reads using sliding window of 110 nt [24]. Candidate miRNA regions contained secondary substructures with the longest unbranched stemloop with at least 20 basepairs. Further, the candidate mature miRNA detected by sequencing did not span the loop: at least 16 nucleotides of candidate mature miRNA sequences fall within the stem structure [10]. If more than one sequence with the same 5' end mapped within the stem-loop structure, we used the highest expressed sequence as the reference candidate mature miRNA [19]. Next, we used miPred to determine the likelihood that the identified candidate miRNA regions are real miRNA precursors, based on their structure and thermodynamic information [25]. Each RNALfold-predicted candidate hairpin was tested against a control set of its 1,000 dinuclotide-randomized sequences, comparing the minimal free energy (MFE) and local contiguous triplet structure composition. Default parameters were used, and the miPred score cut-off was set to 0.70. Besides RNALfold and miPred, we used miRDeep to detect miRNA in the dataset in order to improve the accuracy and robustness of prediction [18]. MiRDeep is based on the miRNA biogenesis probabilistic model, and makes use of the information of position and frequency of candidate mature sequences in the miRNA precursors secondary structure. MiRDeep uses annotated miRNA to estimate the sensitivity of the model, while using 100 permutations of reads and their relative positions within the predicted structure of the candidate miRNA precursor as a control set. All mapped small RNA (reads coverage ≥2) contained in our dataset were used as the input of miRDeep, and miRNA prediction was carried out using default parameters. Candidate miRNA that passed any of the two prediction approaches, but originated within exons of protein-coding genes, were excluded from the following analysis.

### Conversation analysis of known and novel miRNA and their targets

We used phastCon scores based the 17 placental mammal alignment to the human genome to determine the conservation of annotated and candidate mature miRNA sequences [26]. To assess the extent of the conservation, we randomly sampled the same portion of nucleotide from introns and intergenic regions based on composition of candidate mature miRNA sequences. We used LiftOver to convert the phastCon scores to the rhesus macaque genome [46].

For all conserved annotated and candidate mature miRNA (phastCon score ≥0.8) (Additional file 3: Tables S8, Additional file 4: Tables S9, and Additional file 5: Tables S10), we determined sequence conservation of potential target sites. To do so, we used the dataset of human 3' UTR sequences from UCSC genome browser. For each 3' UTR, excluding 15 nt downstream the stop codon, we used 7 nt sliding window with 1 nt step to count different 7-mer motif conservation. Each occurrence of the 7-mer motifs with at least 3 nt having phastCon scores and the mean of phastCon scores larger or equal to 0.8, we classified as conserved. Based on this, we obtained the total frequency and the frequency of the conserved 7-mer motif within 3' UTR regions. For each annotated or candidate mature miRNA, we tested the probability of target site conservation complementary to the miRNA seed region against to the target conservation of the control, using the Binominal test. The control conservation was determined by scrambling the seed region of the tested annotated or candidate miRNA 100 times, based on the same nucleotide composition and similar frequency (±10%) in 3' UTR regions. Thus allowing us to calculate the expected frequency of the 7-mer conservation in 3' UTR regions. Further, we calculated the rank score of the motives based on the phastCon scores in the 7-mer sliding window in 3' UTR sequences as described in [30], using the following formula:

Where t_i _is the total number of the 7-mer occurrences and c_i _is the number of conserved 7-mer occurrences. Next, we sorted t_i _and c_i _by the order from high frequency to low frequency, using the order index as R_ti _and R_ci_, and calculated the score of every motif.

## Authors' contributions

ZY and CM carried out the experiments, NYS, HYH, YX, and HH analyzed the data, NYS, HYH, WC, and PK drafted the manuscript. WC, and PK conceived the study, and participated in its design and coordination. All authors read and approved the final manuscript.

## Supplementary Material

Additional file 1 **Contains Supplemental Supporting Figures and Tables**. Figure S1 - Expression levels of annotated and putative miRNA. Figure S2 - Length distribution of human miRNA. Figure S3 - Expression levels and target site conservation scores of the ncRNA-derived putative conserved miRNA in the human brain. Figure S4 - Expression levels and target site conservation scores of the ncRNA-derived putative conserved miRNA in the human and macaque brain. Figure S5 - Secondary structures, expression and conservation of ncRNA-derived putative conserved miRNA. Table S1 - The samples' age information. Table S2 - The numbers of sequenced and mapped reads. Table S3 - The mapping result and estimation of the false positive mapping rate of the human dataset. Table S4 - The prediction results for annotated miRNA. Table S5 - The prediction results for novel putative miRNA. Table S6 - The prediction results for ncRNA-derived novel putative miRNA. Click here for file

Additional file 2 **Table S7 - Putative human miRNA originating from repeats**. Click here for file

Additional file 3 **Table S8 - Conserved human ncRNA-derived putative miRNA**. Click here for file

Additional file 4 **Table S9 - Conserved annotated human miRNA that pass our prediction approaches**. Click here for file

Additional file 5 **Table S10 - Conserved putative human miRNA (excluding ncRNA-derived miRNA)**. Click here for file
