# Supplementary material for: Comprehensive survey of human brain microRNA by deep sequencing
Source: BMC Genomics. 2010 Jun 30;11:409. doi: 10.1186/1471-2164-11-409 (PMC2996937; doi:10.1186/1471-2164-11-409)
Supplement: Additional file 1 — Contains Supplemental Supporting Figures and Tables. Figure S1 - Expression levels of annotated and putative miRNA. Figure S2 - Length distribution of human miRNA. Figure S3 - Expression levels and target site conservation scores of the ncRNA-derived putative conserved miRNA in the human brain. Figure S4 - Expression levels and target site conservation scores of the ncRNA-derived putative conserved miRNA in the human and macaque brain. Figure S5 - Secondary structures, expression and conservation of ncRNA-derived putative conserved miRNA. Table S1 - The samples' age information. Table S2 - The numbers of sequenced and mapped reads. Table S3 - The mapping result and estimation of the false positive mapping rate of the human dataset. Table S4 - The prediction results for annotated miRNA. Table S5 - The prediction results for novel putative miRNA. Table S6 - The prediction results for ncRNA-derived novel putative miRNA. [file 1471-2164-11-409-S1.DOC]

# Additional file 1

# Supporting Figures


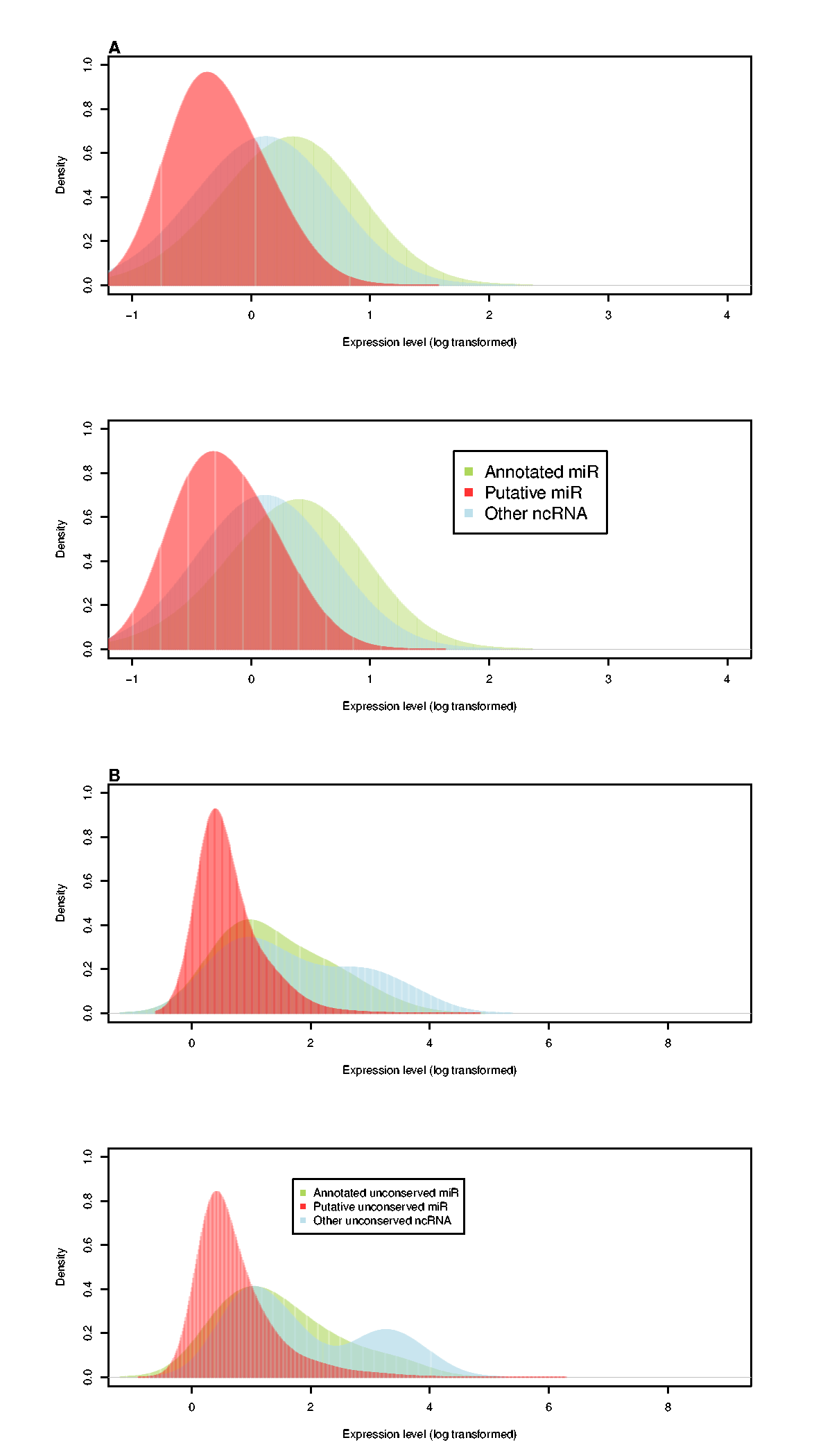


## Figure S1 - Expression levels of annotated and putative miRNA

Distribution of expression levels (base-10 logarithm transformed) of annotated miRNA (Am/green), putative miRNA, excluding the ones originating from ncRNA (Pm/red), and putative miRNA originating from ncRNA (Pn/blue) in humans (upper panel) and rhesus macaques (lower panel) among the total miRNA (**A**) and among non-conserved miRNA (**B**).


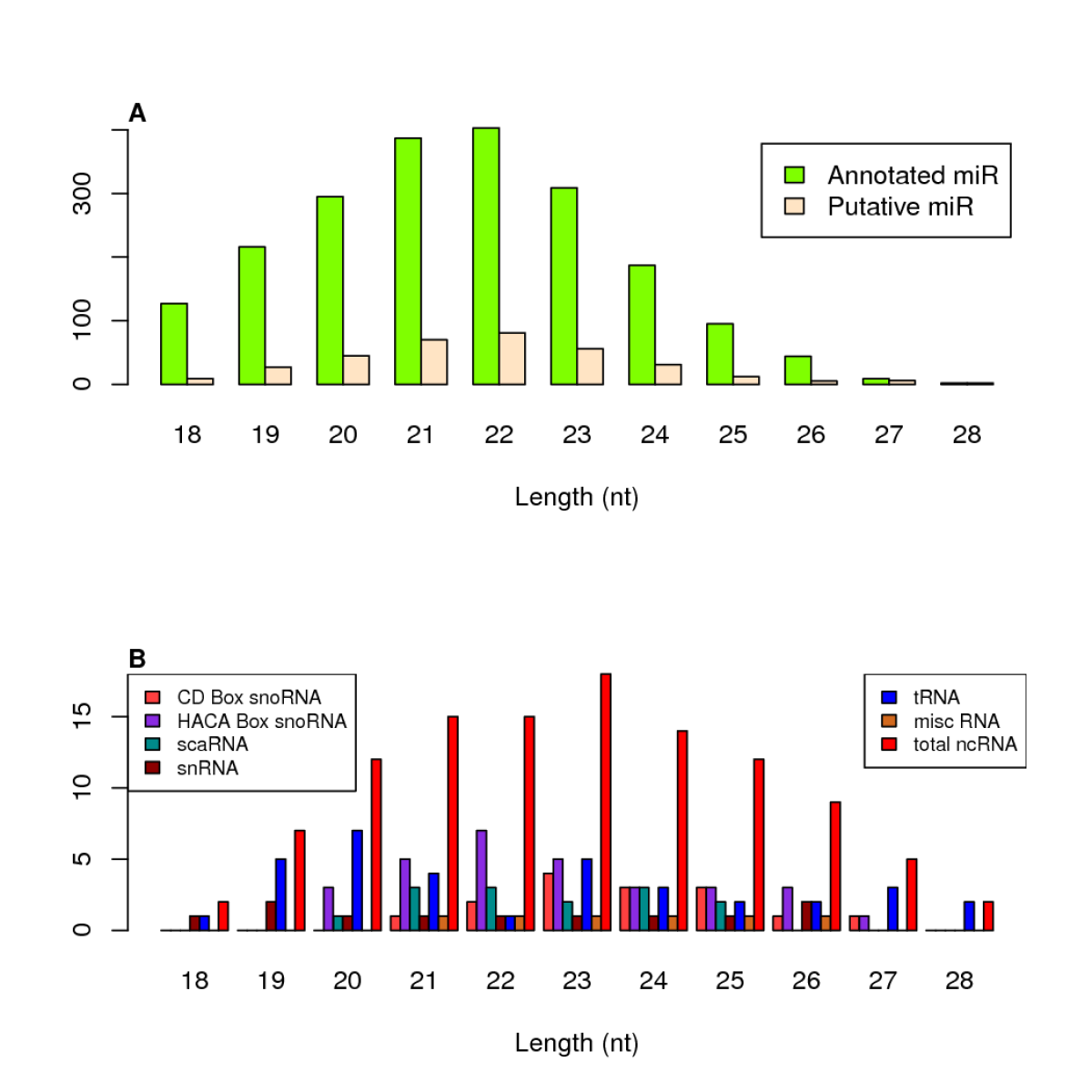


## Figure S2 – Length distribution of human miRNA

The length distribution (in nucleotides) of conserved annotated miRNA (light green), and conserved putative miRNA (light brown) (**A**). The length distribution (in nucleotides) of conserved putative ncRNA-derived miRNA (CD Box RNA: orange; H/ACA Box RNA: purple; scaRNA: dark green; snRNA: dark red; tRNA: green; miscRNA: brown; total: red) (**B**).


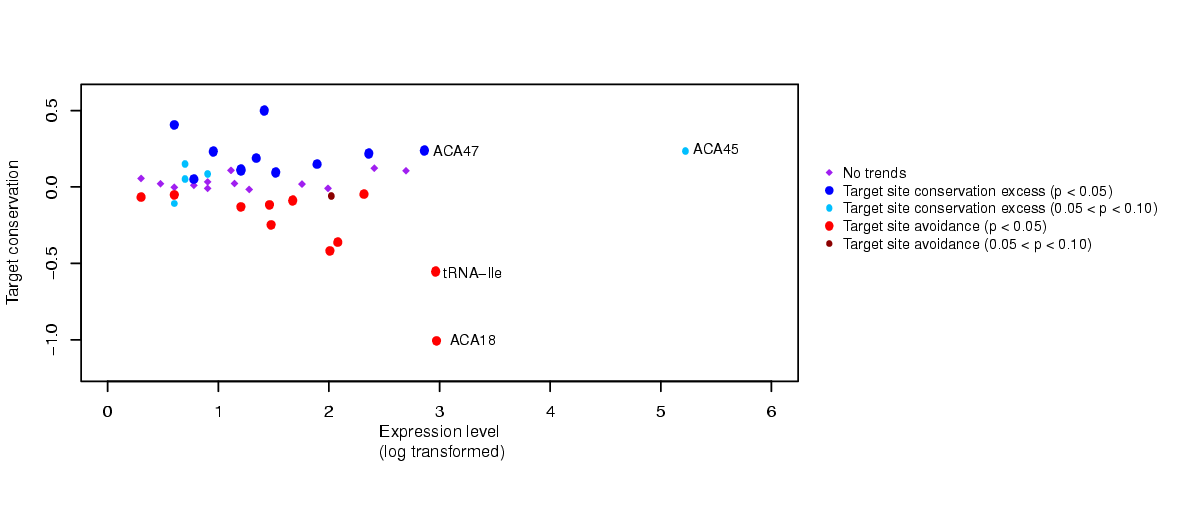


## Figure S3 - Expression levels and target site conservation scores of the ncRNA-derived putative conserved miRNA in the human brain.

Among the total of 41 conserved ncRNA-derived putative miRNA found in the human brain: 11 show significant target site conservation excess (*p*<0.05/blue); 5 show marginally significant excess (0.05<*p*<0.10/lightblue); 11 show significant target avoidance (*p*<0.05/red); 1 shows marginally significant avoidance (0.05<*p*<0.10/brown); the rest shows no significant trends (purple).


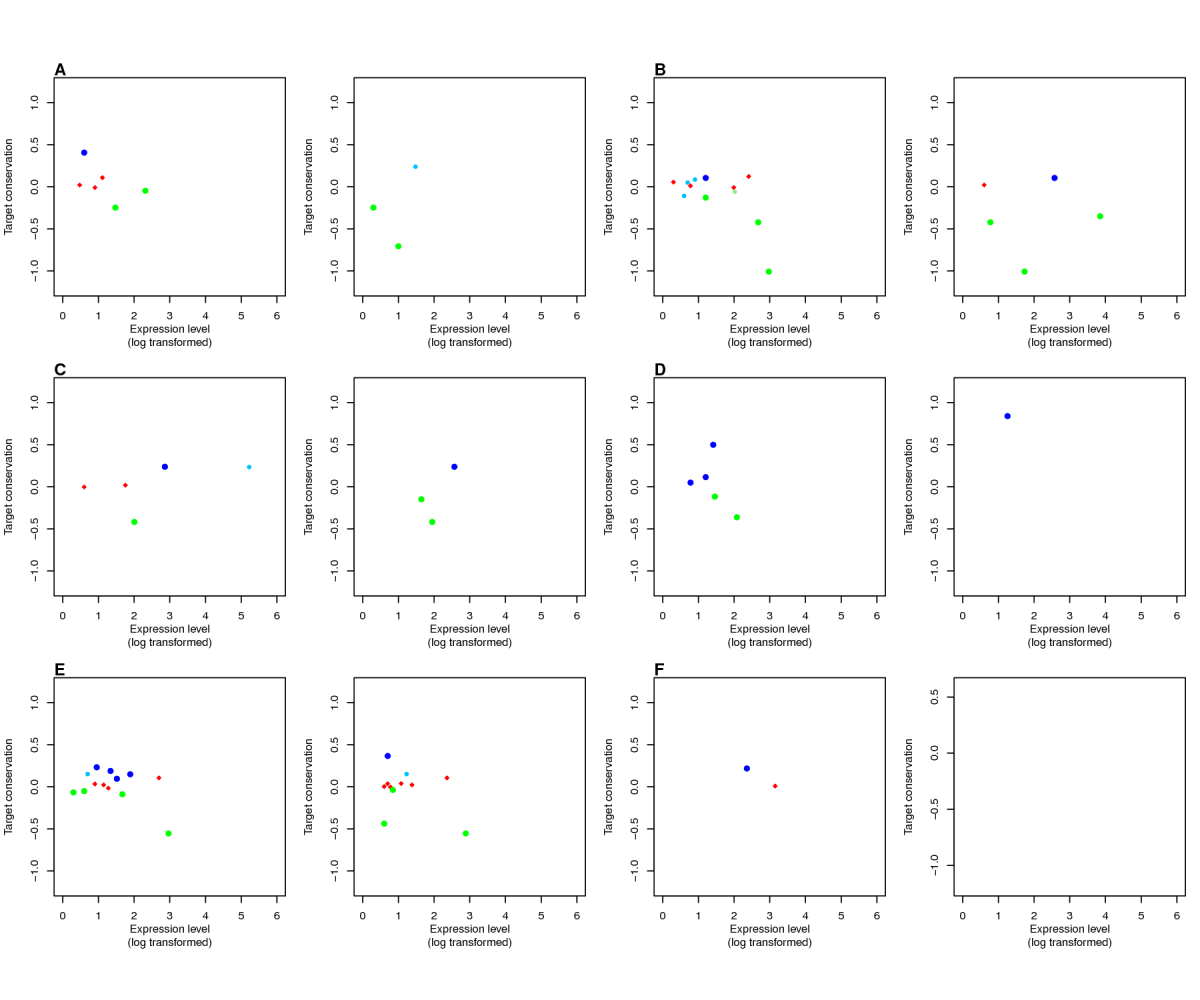


## Figure S4 - Expression levels and target site conservation scores of the ncRNA-derived putative conserved miRNA in the human and macaque brains

The expression level (based-10 logarithm transformed read count) and target conservation of the conserved seed families of putative ncRNA-derived miRNA in humans (left panel) and rhesus macaques (right panel): (**A**) putative miRNA derived from CD Box snoRNA; (**B**) putative miRNA derived from H/ACA Box snoRNA; (**C**) putative miRNA derived from scaRNA snoRNA; (**D**) putative miRNA derived from snRNA; (**E**) putative miRNA derived from tRNA; (**F**) putative miRNA derived from other RNA (Y RNA and SRP RNA). Some seed families showed significant and marginally significant target site conservation excess (*p*<0.05: blue; 0.05<*p*<0.10: light blue). Some seed families showed significant and marginally significant target avoidance (*p*<0.05: green; 0.05<*p*<0.10: grey-green). Seed families with no significant trend are shown in red.


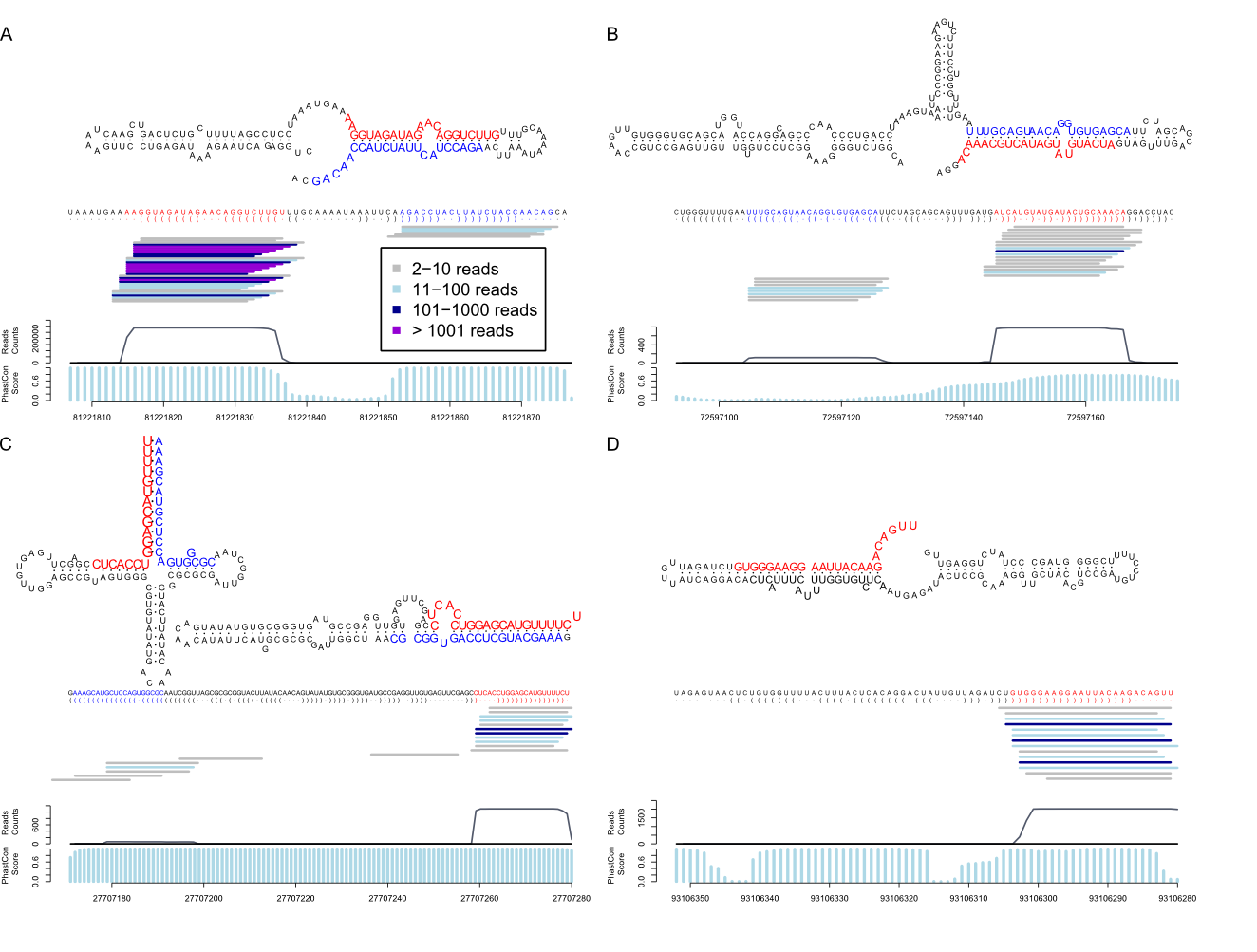


## Figure S5 - Secondary structures, expression and conservation of ncRNA-derived putative conserved miRNA

In each panel shown are: The secondary structure of ncRNA with putative mature miRNA sequence (red), and the putative miRNA-star sequence (blue). The small RNA sequences mapped to the ncRNA and the phastCon scores at each nucleotide position. Displayed are ACA45 RNA (**A**), ACA47 RNA (**B**), tRNA-Ile (**C**), and ACA18 RNA (**D**). For tRNA-Ile two alternative secondary structures are shown.

# Supporting Tables

## Table S1. The samples’ age information

| Human | Age  (days) | 1 | 4 | 34* | 204 | 2922 | 5105 | 9277 | 19213 | 24090 | 29200 | 32120 | 35770* |
| --- | --- | --- | --- | --- | --- | --- | --- | --- | --- | --- | --- | --- | --- |
|  | Age  (years) | 0 | 0 | 0 | 0 | 8 | 13 | 25 | 52 | 66 | 80 | 88 | 98 |
| Rhesus  Macaques | Age  (days) | 16* | 20 | 153 | 207 | 310 | 739 | 1487 | 3389 | 7391 | 8104 | 9518 | 10220* |
|  | Age  (years) | 0 | 0 | 0 | 0 | 0 | 2 | 4 | 9 | 20 | 22 | 26 | 28 |

* These samples we measured in two technical replicates.

## Table S2. The numbers of sequenced and mapped reads *

|  | Total of raw datasets | | Mapped with no mismatches | |
| --- | --- | --- | --- | --- |
|  | Reads | Unique sequences | Reads (mapped/total)*100% | Unique sequences |
| Human | 76,565,933 | 909,917 | 55,061,969 (71.9%) | 106,597 |
| Rhesus  Macaques | 95,326,968 | 970,340 | 69,315,085 (72.7%) | 49,437 |

* Only the sequences with length 18-28 nt represented by at least two sequence reads are listed

## Table S3. The mapping result and estimation of the false positive mapping rate of the human dataset

|  | Real dataset | | | Shuffled dataset | |
| --- | --- | --- | --- | --- | --- |
|  | Reads Number | Unique sequences | Mapping ratio (%) | Unique sequences | False positive ratio |
| Exact mapping | 55,061,969 | 106,597 | 11.7% | 2935.5 | 2.8% |
| Allowing 1-mismatch | 68,515,810 | 227,188 | 25.0% | 61686.7 | 28.4% |
| Allowing 2-mismatch | 73,478,896 | 579,900 | 63.7% | 316948.6 | 65.8% |

**Table S4. The prediction results for annotated miRNA**

|  | Annotated miRNA | Total reads mapped to annotated miRNA | Annotated miR that pass the prediction pipeline | Predicted by miPred | Predicted by miRDeep | Overlap of two approaches |
| --- | --- | --- | --- | --- | --- | --- |
| Human | 602 | 53,567,745 (97.2%) | 523  (86.9%) | 516 | 211 | 204 |
| Rhesus macaque | 493 | 67,893,419 (97.9%) | 423  (85.8%) | 414 | 178 | 169 |

**Table S5. The prediction results for novel putative miRNA ***

|  | Putative miR | New star of annotated miR | Putative novel miR | Predicted by miPred | Predicted by miRDeep | Overlap of two approaches |
| --- | --- | --- | --- | --- | --- | --- |
| Human | 1,433 | 96 | 1,337 | 1,326 | 57 | 46 |
| Rhesus macaque | 1,095 | 73 | 1,022 | 992 | 104 | 74 |

* Excluding ncRNA–derived miRNA.

Table S6. The prediction results for ncRNA-derived novel putative miRNA

|  | Putative miR derived from ncRNA | Predicted by miPred | Predicted by miRDeep | Overlap of two approaches |
| --- | --- | --- | --- | --- |
| Human | 65 | 62 | 8 | 5 |
| Rhesus macaque | 32 | 30 | 4 | 2 |
